# Short-Course Radiotherapy-Based Total Neoadjuvant Therapy plus Tislelizumab for Locally Advanced Rectal Cancer (Neo-STAR): Early Outcomes of a Randomized Phase II Trial

**DOI:** 10.34133/cancomm.0041

**Published:** 2026-07-23

**Authors:** Fengpeng Wu, Xuhua Hu, Baokun Li, Jianfeng Zhang, Guanglin Wang, Haiyan Fan, Guangquan An, Bin Yu, Hongqing Ma, Botian Zhao, Zhihan Li, Bo Gao, Ming Liu, Xuan Wang, Dan Liu, Jitao Hu, Hui Liu, Youqiang Liu, Feifei Wang, Juan Zhang, Jun Feng, Xiaoran Wang, Zesong Meng, Zhenya Zhang, Zheng Li, Jingyi Sun, Shihao Liu, Na Wang, Jing Han, Wenbo Niu, Chaoxi Zhou, Linlin Xiao, Guiying Wang

**Affiliations:** ^1^ Department of Radiation Oncology, the Fourth Hospital of Hebei Medical University, Shijiazhuang, Hebei 050011, P. R. China.; ^2^ The Second Department of Surgery, the Fourth Hospital of Hebei Medical University, Shijiazhuang, Hebei 050011, P. R. China.; ^3^ Department of Gastroenterology, the Fourth Hospital of Hebei Medical University, Shijiazhuang, Hebei 050011, P. R. China.; ^4^ Department of General Surgery, the Second Hospital of Hebei Medical University, Shijiazhuang, Hebei 050004, P. R. China.; ^5^ Cancer Institute, the Fourth Hospital of Hebei Medical University, Shijiazhuang, Hebei 050011, P. R. China.; ^6^ Department of Medical Oncology, the Fourth Hospital of Hebei Medical University, Shijiazhuang, Hebei 050011, P. R. China.

## Abstract

**Background:** Short-course radiotherapy (SCRT)-based total neoadjuvant therapy (TNT) is used for locally advanced rectal cancer (LARC). However, the pathological complete response (pCR) rate still hovers around 30%. Radiotherapy and immune checkpoint inhibitors have been shown to exert synergistic anticancer effects. This phase II randomized clinical trial aimed to evaluate the efficacy and safety of SCRT followed by capecitabine plus oxaliplatin (CAPOX) and tislelizumab versus SCRT followed by CAPOX alone in LARC. **Methods:** Patients initially diagnosed with clinical tumor stage 1 to 2, with node involvement and no distant metastasis (cT_1-2_N_+_M_0_) or clinical tumor stage 3 to 4, with any node status and no distant metastasis (cT_3-4_N_any_M_0_) rectal adenocarcinoma were randomly assigned to receive SCRT (25 Gy in 5 fractions [25 Gy/5F]), followed by 4 cycles of CAPOX combined with tislelizumab (SCRT-TNT-ICI) or CAPOX alone (SCRT-TNT). After total mesorectal excision, 2 cycles of postoperative chemotherapy were administered according to the patient’s preference. The primary end point was the pCR rate, and secondary end points included major pathological response (tumor regression grade 0 or 1), 3-year progression-free survival, 3-year overall survival, and adverse events. **Results:** Between September 2021 and March 2024, 118 patients were randomized, of whom 111 started the allocated treatment, with 53 and 58 in SCRT-TNT-ICI and SCRT-TNT groups, respectively. Of those, 89 patients had surgical resection, including 45 in the SCRT-TNT-ICI group and 44 in the SCRT-TNT group. The pCR rate was 45.3% (95% confidence interval [CI], 31.5% to 59.8%) in the SCRT-TNT-ICI group compared to 27.6% (95% CI, 16.6% to 40.8%) in the SCRT-TNT group (odds ratio = 2.17; 95% CI, 0.99 to 4.79; *P* = 0.052). The major pathological response rates were 50.9% (95% CI, 36.6% to 65.2%) and 31.0% (95% CI, 19.5% to 44.6%), respectively (odds ratio = 2.31; 95% CI, 1.06 to 5.01; *P* = 0.033). During the neoadjuvant treatment period, the incidence of grade 3 to 4 adverse events was comparable between the SCRT-TNT-ICI and SCRT-TNT groups, with anemia being the most common in both groups. **Conclusion:** This phase II study provides preliminary evidence of promising tumor regression with SCRT-TNT combined with tislelizumab in LARC, warranting further validation in phase III trials. **Trial registration:** This trial was registered at clinicaltrials.gov (Identifier: NCT05086627).

## Background

According to current National Comprehensive Cancer Network guidelines, total neoadjuvant therapy (TNT) followed by total mesorectal excision (TME) constitutes the standard treatment for locally advanced rectal cancer (LARC). The TNT strategy markedly improves the pathological complete response (pCR) and reduces the risk of distant metastasis compared with the traditional neoadjuvant chemoradiotherapy or radiotherapy approach, thereby improving patient survival [1–4]. Nevertheless, the pCR rates reported for these studies still hover around 30%. Currently, there is an urgent need to further improve the tumor regression rate to increase sphincter preservation rates and enhance patients’ quality of life.

Immune checkpoint inhibitors (ICIs) exert remarkable effects in metastatic and locally advanced colorectal cancer with deficient mismatch repair/microsatellite instability-high (MSI-H) [5–8]. However, administering ICIs alone in patients with proficient mismatch repair/microsatellite stable/microsatellite instability-low does not result in ideal outcomes [7]. Previous studies have demonstrated that the combination of radiotherapy and ICIs could result in a synergistic anticancer response in other malignant tumors [9,10]. Subsequent phase II to III clinical trials have also confirmed the efficacy of radiotherapy combined with ICIs in LARC patients with proficient mismatch repair/microsatellite stable/microsatellite instability-low [11–19]. Multiple studies have reported pCR rates exceeding 30%, and the combination of short-course radiotherapy (SCRT) with ICIs appears to be more effective [11,12,15,18,19]. For instance, in the UNION phase III, multicenter randomized clinical trial (RCT), neoadjuvant SCRT followed by camrelizumab plus capecitabine plus oxaliplatin (CAPOX) achieved a significantly higher pCR rate (39.8%) compared with long-course chemoradiotherapy (LCRT) followed by CAPOX alone (15.3%) [12].

To date, there has been no clinical study comparing SCRT-based TNT plus ICIs (SCRT-TNT-ICI) with standard SCRT-TNT. Therefore, we conducted this prospective, phase II, RCT to explore whether the incorporation of ICIs into SCRT-TNT could further enhance tumor regression and survival outcomes in this patient population.

## Materials and Methods

### Study design and participants

This study (the Neo-STAR trial) was a prospective phase II RCT conducted at the Fourth Hospital of Hebei Medical University (Ethics approval number: 2021104), designed to assess the efficacy of SCRT-TNT with or without ICIs for LARC. This investigator-initiated trial, carried out in accordance with the ethical standards of the World Medical Association’s Declaration of Helsinki, was registered at ClinicalTrials.gov (identifier: NCT05086627). All participants provided written informed consent.

The major inclusion criteria were as follows: 18 to 75 years old; Eastern Cooperative Oncology Group performance status (ECOG PS) of 0 or 1; tumor location within 10 cm of the anal verge; initial diagnosis of rectal cancer patients with clinical tumor stage 1 to 2, with node involvement and no distant metastasis (cT_1-2_N_+_M_0_) or clinical tumor stage 3 to 4, with any node status and no distant metastasis (cT_3-4_N_any_M_0_) according to the American Joint Committee on Cancer 8th edition; regardless of mismatch repair (MMR)/microsatellite instability (MSI) status; adequate organ function. The main exclusion criteria included the following: a history of antitumor treatment; a history of allergy to a study drug; required treatment with corticosteroids (dose equivalent to prednisone of >10 mg/d) or other immunosuppressive agents within 2 weeks prior to study drug administration; pregnancy or breastfeeding in women. The full inclusion and exclusion criteria are further detailed in the Supplementary Materials.

### Randomization

Randomization was performed centrally at the Clinical Trials Center of the Fourth Hospital of Hebei Medical University. A computer-generated allocation sequence was created using permuted blocks (block size of 8) with a 1:1 allocation ratio. The block structure was concealed from all investigators and participants, being known only to the study coordinator and statistician. While investigators and patients were aware of the treatment assignments, the outcome assessors (including radiologists, pathologists, and statisticians not involved in patient care) remained blinded to the group allocation throughout the trial. No stratification factors were prespecified.

### Procedures and study treatment regimens

In the present study, patients were randomly assigned to the SCRT-TNT-ICI or SCRT-TNT group. Both groups received SCRT (25 Gy in 5 fractions [25 Gy/5F]), followed 3 to 7 d later by 4 cycles of CAPOX chemotherapy and TME surgery 3 weeks after completing chemotherapy. In addition to this regimen, patients in the SCRT-TNT-ICI group received an intravenous infusion of tislelizumab (200 mg) on the first day of each CAPOX cycle. Intensity-modulated radiation therapy was employed for irradiation. The clinical target volume encompassed the primary tumor, metastatic lymph nodes, and regional lymphatic drainage areas [20,21]. The planning target volume was defined as the clinical target volume expanded outward by 0.3 cm. The CAPOX regimen consisted of capecitabine 1,000 mg/m^2^ orally twice daily on days 1 to 14 combined with oxaliplatin 130 mg/m^2^ intravenously on day 1, repeated every 3 weeks. The administration of postoperative chemotherapy was primarily based on patient preference, and when given, 2 cycles of CAPOX were administered.

Adverse events (AEs) were closely monitored and actively managed during the SCRT process and the resting period thereafter, the entire preoperative systemic treatment, the resting period after the TME surgery, and the postoperative chemotherapy period for patients who elected to receive it. AEs were assessed by National Cancer Institute Common Terminology Criteria for Adverse Events Version 5.0 [22]. Postoperative complications were graded according to the Clavien–Dindo classification system [23]. Immunotherapy-related AEs were managed in accordance with the National Comprehensive Cancer Network clinical practice guidelines in oncology for management of immunotherapy-related toxicities (version 3.2021) [24].

The primary end point was the pCR rate, defined as the proportion of patients achieving ypT_0_N_0_M_0_. Secondary end points included major pathological response (MPR, tumor regression grade [TRG] of 0 or 1), 3-year progression-free survival (PFS, defined as the time from randomization to disease progression, recurrence, or death from any cause), 3-year overall survival (OS), defined as the time from randomization to death from any cause), and AEs.

### Data collection

The imaging and laboratory data of all enrolled patients were collected. Tumor imaging assessments were performed by abdominal and pelvic contrast-enhanced magnetic resonance imaging, chest computed tomography, and colonoscopy. Immunohistochemistry was conducted to assess the expression of programmed death-ligand 1 (PD-L1) and DNA MMR proteins (MutS Homolog 6 (MSH6), MutS Homolog 2 (MSH2), MutL Homolog 1 (MLH1), and PMS1 Homolog 2 (PMS2). Genetic testing was performed to determine MSI status in patients. Tumor imaging assessments were conducted before the first and third cycles of chemotherapy with/without ICIs and before surgery. The patients were followed up every 3 months for the first 3 years, every 6 months at 3 to 5 years, and annually thereafter until disease progression/recurrence, loss to follow-up, or death.

### Assessments

Postoperative pathological assessment was independently performed by 2 experienced pathologists using the American Joint Committee on Cancer 8th edition TRG system. TRG was defined as follows: 0, no remaining viable cancer cells (complete response); 1, a single cell or small groups of cancer cells (moderate response); 2, residual cancer outgrown by fibrosis (minimal response); 3, minimal or no tumor cell death, with extensive residual cancer (poor response). In cases where MSI and MMR tests yield inconsistent results, the outcome of the MSI test should be given priority consideration.

### Statistical analysis

Given that the treatment regimen of the SCRT-TNT-ICI group in this study was similar to that of Lin et al. [11], which achieved 48.15% pCR, and the SCRT-TNT group basically had a treatment regimen consistent with the trial group of STELLAR [3], which achieved 16.16% pCR; we expected that the pCR rates of the SCRT-TNT-ICI and SCRT-TNT groups in this study would be 49% and 17%, respectively. Assuming a 2-sided type I error rate (α) of 0.05 and a power of 0.9, the required sample size was calculated using the PASS sample size software (version 15.0) to be 100 cases, with 50 cases assigned to each group. Anticipating a dropout rate of 15%, a total of 118 patients were needed.

Effectiveness analyses were performed on the modified intention-to-treat (mITT) and per protocol set (PPS) populations. The mITT population included all patients who initiated the allocated treatment, excluding those who withdrew prior to receiving any treatment. The PPS population consisted of patients who completed the prespecified treatment protocol, including neoadjuvant therapy and surgical treatment. Safety analyses were conducted on the mITT population. Missing data were handled as follows: for the primary end point (pCR), missing data points were considered as non-pCR events; multiple imputation was utilized for continuous variables, and standard censoring was used for time-to-event data.

Comparisons of pCR and MPR rates between the 2 groups were performed using the chi-square test. Between-group comparisons of AEs were performed using Fisher’s exact test. The Clopper–Pearson method was used to calculate 95% confidence intervals (CIs) for pCR and MPR rates. The covariates selected were age, sex, ECOG PS, PD-L1 combined positive score (CPS), MMR status, clinical T stage, clinical N stage, overall clinical stage, distance from the primary tumor to the anal verge, extramural vascular invasion (EMVI), and mesorectal fascia. *P* < 0.05 was considered statistically significant.

For time-to-event end points, including PFS and OS, survival curves, median survival times, and specific-time survival rates were estimated by the Kaplan–Meier method. Comparisons of survival curves between the 2 treatment arms were performed by a 2-sided stratified log-rank test. Furthermore, a Cox proportional hazards model was employed to estimate the hazard ratio (HR), with treatment arm as the sole covariate and stratification based on stratum factors. All analyses were performed with the SPSS software 27.0 (SPSS, Inc.).

## Results

### Patient characteristics

**Fig. 1.**
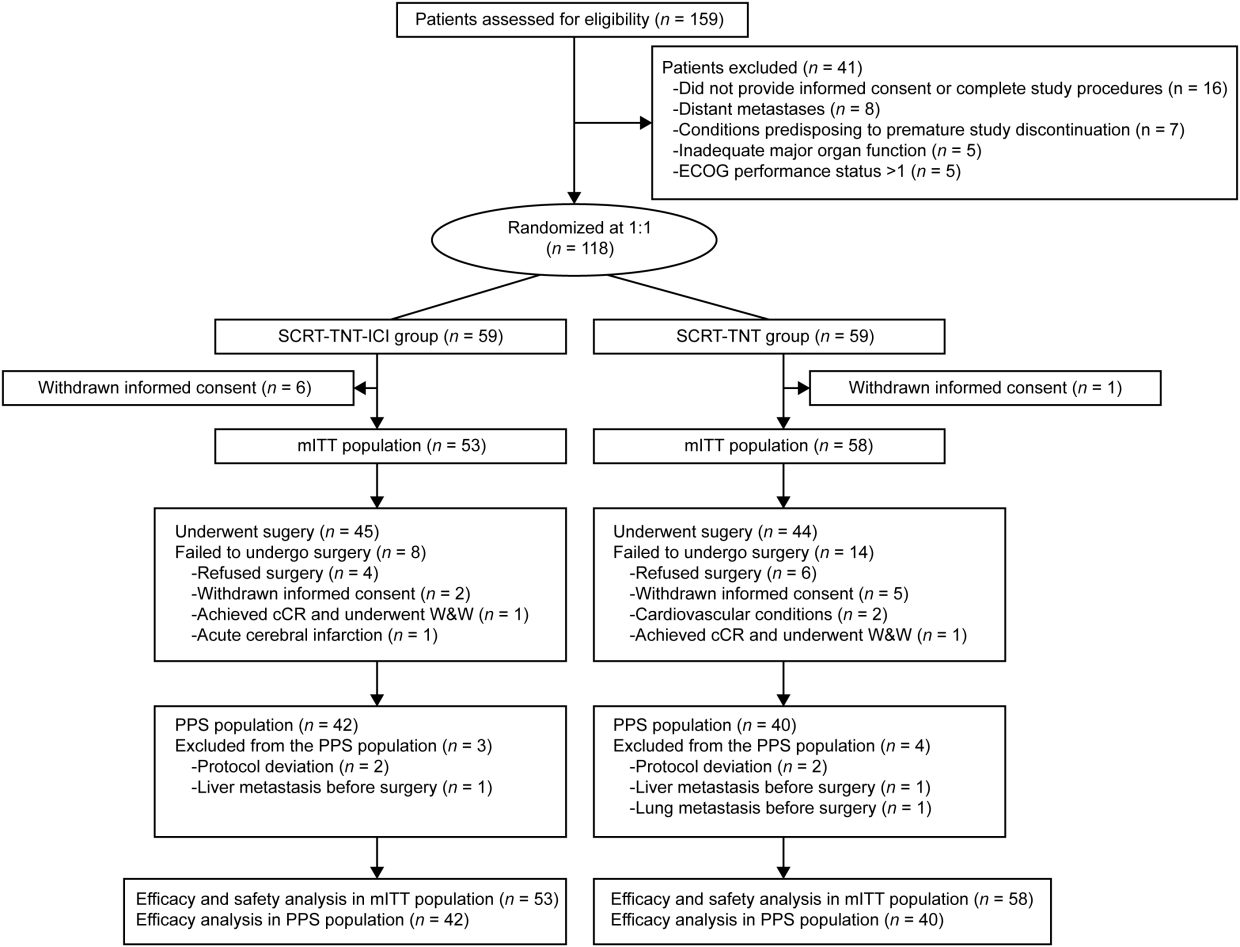
Study flowchart. mITT, modified intention-to-treat; PPS, per protocol set; cCR, clinical complete response; W&W, watch and wait.

Between 2021 September 1 and 2024 March 15, a total of 118 patients who met the eligibility criteria were randomized to the SCRT-TNT-ICI group (*n* = 59) and SCRT-TNT group (*n* = 59) (Fig. 1). Six patients in the SCRT-TNT-ICI group and 1 in the SCRT-TNT group withdrew after randomization but prior to the initiation of treatment. As a result, the mITT population consisted of 53 patients in the SCRT-TNT-ICI group and 58 patients in the SCRT-TNT group, and their baseline characteristics are detailed in Table 1.

**Table 1.** Baseline characteristics of the mITT population (*n* = 111)

Characteristics	SCRT-TNT-ICI group (*n* = 53)	SCRT-TNT group (*n* = 58)
Sex		
Male	37 (69.8%)	45 (77.6%)
Female	16 (30.2%)	13 (22.4%)
Age, years		
Median (range)	55 (30 to 74)	65 (36 to 75)
Age category, years		
≤60	36 (67.9%)	20 (34.5%)
>60	17 (32.1%)	38 (65.5%)
ECOG PS		
0	27 (50.9%)	27 (46.6%)
1	26 (49.1%)	31 (53.4%)
PD-L1 CPS		
<1	14 (26.4%)	18 (31.0%)
≥1	27 (50.9%)	26 (44.8%)
Unknown	12 (22.6%)	14 (24.1%)
MMR status		
dMMR or MSI-H ^a^	3 (5.7%)	1 (1.7%)
pMMR or MSI-L or MSS	50 (94.3%)	56 (96.6%)
Unknown	0 (0.0%)	1 (1.7%)
Clinical T stage		
cT2	2 (3.8%)	3 (5.2%)
cT3	35 (66.0%)	36 (62.1%)
cT4	16 (30.2%)	19 (32.8%)
Clinical N stage		
N0	3 (5.7%)	3 (5.2%)
N1	15 (28.3%)	17 (29.3%)
N2	35 (66.0%)	38 (65.5%)
Clinical stage		
II	3 (5.7%)	2 (3.4%)
III	50 (94.3%)	56 (96.6%)
Distance from primary tumor to anal verge, cm		
≤5	35 (66.0%)	36 (62.1%)
5 to 10	18 (34.0%)	22 (37.9%)
EMVI		
Positive	24 (45.3%)	24 (41.4%)
Negative	29 (54.7%)	34 (58.6%)
MRF		
Positive	26 (49.1%)	32 (55.2%)
Negative	27 (50.9%)	26 (44.8%)

ECOG PS, Eastern Cooperative Oncology Group performance status; PD-L1 CPS, programmed cell death-ligand 1 combined positive score; MMR, mismatch repair; dMMR, deficient mismatch repair; pMMR, proficient mismatch repair; MSI-H, microsatellite instability-high; MSI-L, microsatellite instability-low; MSS, microsatellite stable; EMVI, extramural vascular invasion; MRF, mesorectal fascia; mITT, modified intention-to-treat

^a^ There was 1 patient with MSI-H (pMMR) in the SCRT-TNT-ICI group.

A total of 89 (80.2%) patients in the mITT (*n* = 111) population received surgical intervention: 45 (84.9%) of 53 in the SCRT-TNT-ICI group and 44 (75.9%) of 58 in the SCRT-TNT group. The specific reasons for omitting surgery are summarized in the study flowchart (Fig. 1). In the SCRT-TNT-ICI group, 8 patients did not undergo surgery: 4 refused, 2 withdrew consent, 1 achieved clinical complete response (cCR) and entered a Watch & Wait protocol, and 1 experienced an acute cerebral infarction. In the SCRT-TNT group, 14 patients did not receive surgery: 6 refused, 5 withdrew informed consent, 2 had cardiovascular conditions, and 1 entered Watch & Wait after attaining cCR. Among the 89 patients who received surgery, 82 patients (42 in the SCRT-TNT-ICI group and 40 in the SCRT-TNT group) completed the prespecified treatment protocol and were included in the PPS population. Of the 7 patients excluded from the PPS population due to protocol deviation, 4 (2 patients in each group) received an additional cycle of neoadjuvant chemotherapy due to delayed surgery. In the SCRT-TNT-ICI group, 1 patient was found to have liver metastasis during the preoperative evaluation after neoadjuvant therapy. In the SCRT-TNT group, 1 patient was diagnosed with lung metastasis during preoperative assessment, and another patient was identified with liver metastasis on restaging following the second cycle of neoadjuvant chemotherapy.

Excluding the 3 patients who developed distant metastases during the neoadjuvant phase, postoperative CAPOX was administered to 65.9% (29/44) of patients in the SCRT-TNT-ICI group and 59.5% (25/42) in the SCRT-TNT group. The median interval from SCRT completion to TME was 17.79 (interquartile range, 16.21 to 19.25) weeks in the SCRT-TNT-ICI group and 17.50 (interquartile range, 16.43 to 20.07) weeks in the SCRT-TNT group.

### Efficacy

In the mITT population, 45.3% (95% CI, 31.5% to 59.8%) of the SCRT-TNT-ICI group achieved pCR, versus 27.6% (95% CI, 16.6% to 40.8%) in the SCRT-TNT group (odds ratio [OR] = 2.17; 95% CI, 0.99 to 4.79; *P* = 0.052). MPR rates in the SCRT-TNT-ICI and SCRT-TNT groups were 50.9% (95% CI 36.6% to 65.2%) and 31.0% (95% CI, 19.5% to 44.6%) (OR = 2.31; 95% CI, 1.06 to 5.01; *P* = 0.033), respectively (Table 2). Additionally, 1 patient each in the SCRT-TNT-ICI and SCRT-TNT groups achieved cCR and did not undergo surgery.

**Table 2.** The surgical and postoperative pathological outcomes

Groups	SCRT-TNT-ICI	SCRT-TNT
mITT population		
Sample size, *n*	53	58
pCR, *n* (%; 95% CI)	24 (45.3; 31.5%–59.8%)	16 (27.6; 16.6%–40.8%)
MPR, *n* (%; 95% CI)	27 (50.9; 36.6%–65.2%)	18 (31.0; 19.5%–44.6%)
Surgery population		
Sample size, *n*	45	44
R0 resection, *n* (%)		
Yes	45 (100.0) ^a^	44 (100.0) ^b^
No	0 (0.0)	0 (0.0)
TRG score, *n* (%)		
0	24 (53.3)	17 (38.6)
1	3 (6.7)	2 (4.5)
2	16 (35.6)	16 (36.4)
3	2 (4.4)	9 (20.5)
PPS population		
Sample size, *n*	42	40
pCR, *n* (%; 95% CI)	23 (54.8; 39.1%–69.7%)	15 (37.5; 24.2%–53.2%)
MPR, *n* (%; 95% CI)	26 (61.9; 48.2%–74.2%)	17 (42.5; 26.6%–59.2%)
R0 resection, *n* (%)		
Yes	42 (100.0)	40 (100.0)
No	0 (0.0)	0 (0.0)
TRG score, *n* (%)		
0	23 (54.8)	15 (37.5)
1	3 (7.1)	2 (5.0)
2	14 (33.3)	15 (37.5)
3	2 (4.8)	8 (20.0)
Pathological T stage, *n* (%)		
ypT0	24 (57.1)	15 (37.5)
ypTis	1 (2.4)	0 (0.0)
ypT1	0 (0.0)	1 (2.5)
ypT2	9 (21.4)	6 (15)
ypT3	6 (14.3)	10 (25.0)
ypT4	2 (4.8)	8 (20.0)
Pathological N stage, *n* (%)		
ypN0	39 (92.9)	36 (90.0)
ypN1	3 (7.1)	3 (7.5)
ypN2	0 (0.0)	1 (2.5)
Type of surgery, *n* (%)		
Anterior resection	36 (85.7)	24 (60.0)
Abdominoperineal resection	6 (14.3)	13 (32.5)
Hartmann procedure	0 (0.0)	3 (7.5)
Anal sphincter preservation, *n* (%)		
Yes	36 (85.7)	27 (67.5)
No	6 (14.3)	13 (32.5)

mITT, modified intention-to-treat; PPS, per protocol set; pCR, pathological complete response; MPR, major pathological response; TRG, tumor regression grade.

^a^ One patient had liver metastasis before surgery.

^b^ One patient had liver metastasis, and 1 patient had lung metastasis before surgery.

**Fig. 2.**
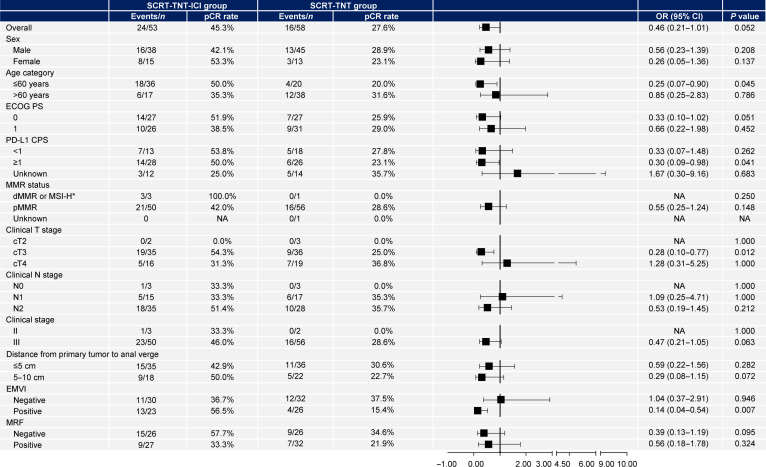
Forest plot with differences for pathological complete response rates in subgroups. ECOG PS, Eastern Cooperative Oncology Group performance status; PD-L1 CPS, programmed cell death-ligand 1 combined positive score; MMR, mismatch repair; dMMR, deficient mismatch repair; pMMR, proficient mismatch repair; EMVI, extramural vascular invasion; MRF, mesorectal fascia; pCR, pathological complete response; MPR, major pathological response. *There was 1 patient with microsatellite instability-high (MSI-H) (pMMR) in the SCRT-TNT-ICI group.

To evaluate the impact of baseline characteristics on pCR outcomes between the 2 regimens, subgroup analyses were performed across 11 variables, including sex, age, ECOG PS, EMVI, and PD-L1 expression, among others. Subgroup analysis identified that the following patient characteristics were associated with a significantly higher pCR rate following SCRT-TNT-ICI: age ≤60 years (OR = 0.25; 95% CI, 0.07 to 0.90; *P* = 0.045), PD-L1 CPS ≥1 (OR = 0.30; 95% CI, 0.09 to 0.98; *P* = 0.041), cT3 stage (OR = 0.28; 95% CI, 0.10 to 0.77; *P* = 0.012), and positive EMVI (OR = 0.14; 95% CI, 0.04 to 0.54; *P* = 0.007) (Fig. 2).

Due to a significant difference in baseline age between the 2 patient groups, we conducted a propensity score-matching analysis and successfully matched 27 pairs of patients (Table S1). The results showed that pCR rates were 55.6% (95% CI, 35.4% to 74.5%) and 29.6% (95% CI, 13.8% to 49.4%) in the SCRT-TNT-ICI group and SCRT-TNT group (*P* = 0.052), respectively. MPR rates were 63.0% (95% CI, 42.5% to 80.6%) and 33.3% (95% CI, 16.5% to 54%) in the 2 groups (*P* = 0.029), respectively (Table S2).

**Fig. 3.**
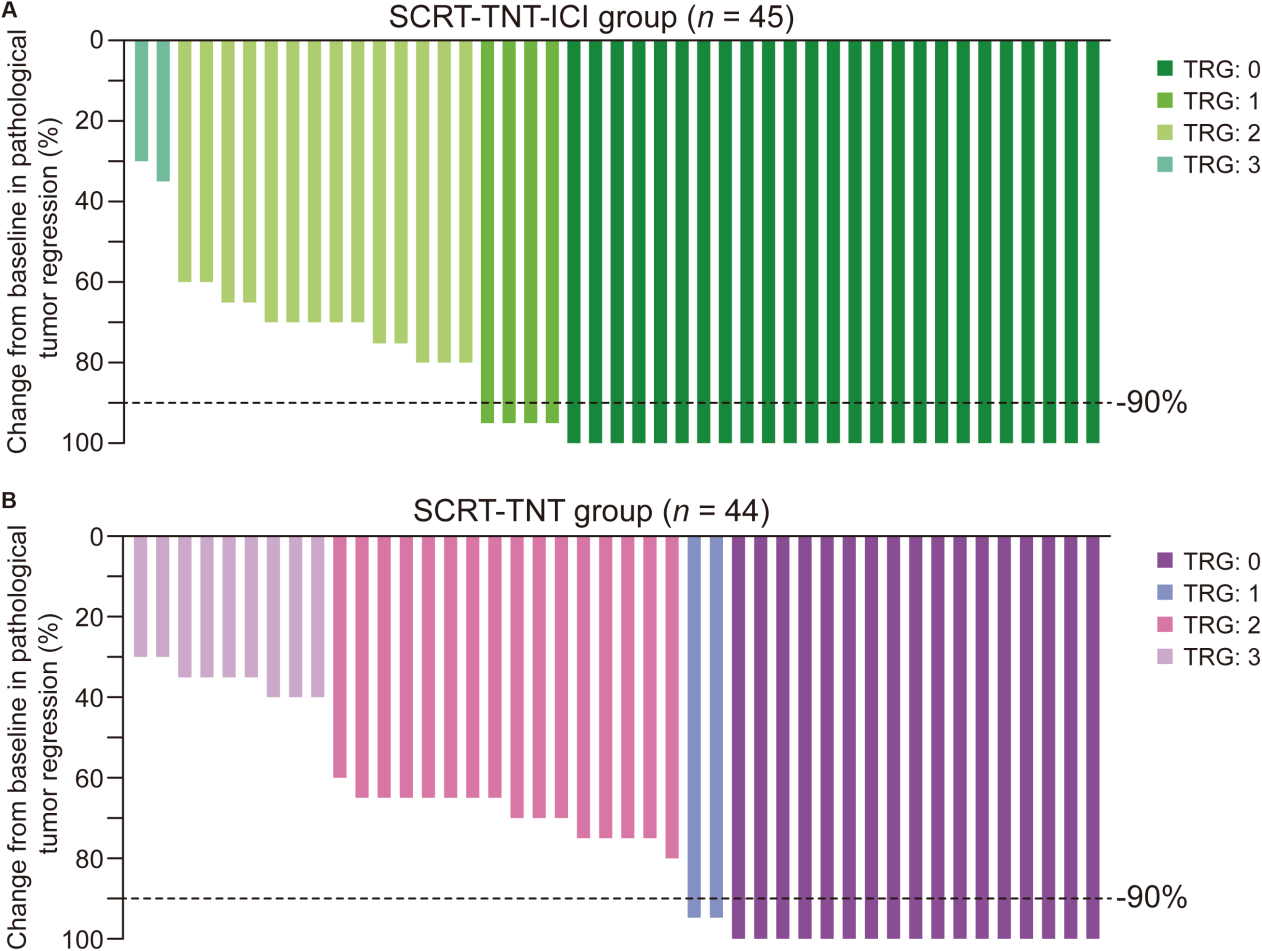
Pathological regression of the primary tumor. (A) SCRT-TNT-ICI group. (B) SCRT-TNT group.

In the PPS population, all 82 patients administered surgery achieved R0 resection (Table 2). The pCR rates of the SCRT-TNT-ICI group and the SCRT-TNT group were 54.8% (95% CI, 39.1% to 69.7%) and 37.5% (95% CI, 24.2% to 53.2%), respectively. The MPR rates were 61.9% (95% CI, 48.2% to 74.2%) and 42.5% (95% CI, 26.6% to 59.2%) in the SCRT-TNT-ICI and SCRT-TNT groups, respectively. Sphincter-sparing surgery was performed in 36 (85.7%) patients in the SCRT-TNT-ICI group and 27 (67.5%) patients in the SCRT-TNT group. Notably, the SCRT-TNT-ICI group demonstrated deeper tumor regression, with a higher proportion of TRG 0 (54.8% versus 37.5%) and a lower proportion of TRG 3 (4.8% versus 20.0%) compared with the SCRT-TNT group (Table 2 and Fig. 3). This translated into more favorable downstaging, as evidenced by a higher ypT0 rate (57.1% versus 37.5%) and a lower rate of residual ypT3–4 disease (19.1% versus 45.0%).

**Fig. 4.**
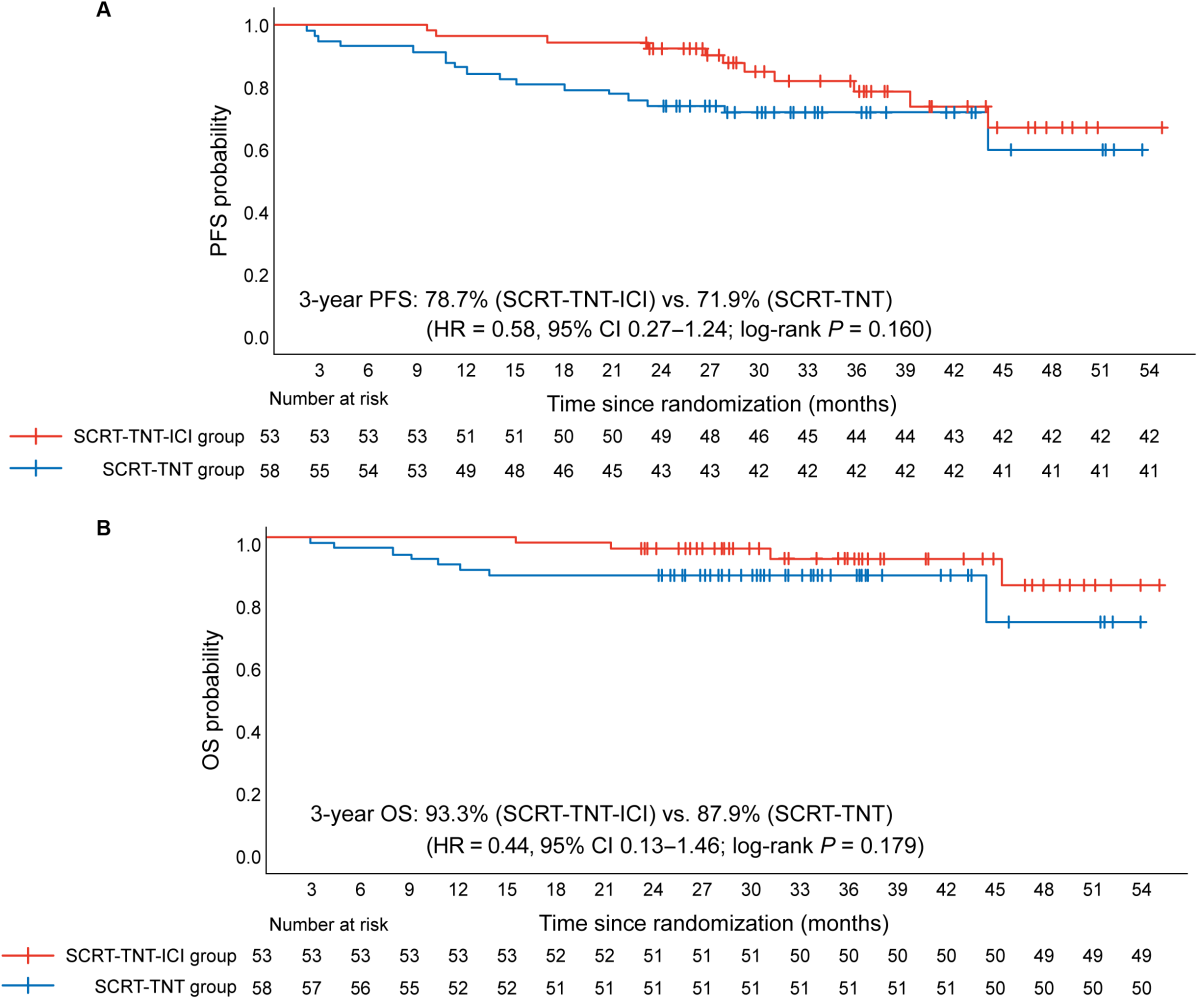
Kaplan–Meier estimates of survival in the SCRT-TNT-ICI group (red line) and SCRT-TNT group (blue line). (A) Progression-free survival (PFS). (B) Overall survival (OS). HR, hazard ratio; CI, confidence interval.

At the cutoff date for this analysis (2026 February 25), the median follow-up for the whole cohort was 33.74 months (range 23.06 to 54.73 months). After follow-up and survival analysis, the 3-year PFS rates were 78.7% and 71.9% in the SCRT-TNT-ICI and SCRT-TNT groups (HR = 0.58, 95% CI, 0.27 to 1.24; *P* = 0.160), and the 3-year OS rates were 93.3% and 87.9% (HR = 0.44, 95% CI, 0.13 to 1.46; *P* = 0.179), respectively (Fig. 4).

### Safety

During the neoadjuvant treatment period, grade 3 to 4 AEs occurred in 9.4% (5/53) and 10.3% (6/58) of the SCRT-TNT-ICI and SCRT-TNT groups, respectively (Table 3). Anemia was the most common grade 3 to 4 AE (5.7% versus 6.9%), with no significant difference in hematologic toxicity between groups. Hypothyroidism (grade 1 to 2) was more frequent in the SCRT-TNT-ICI group (11.3% versus 1.7%; *P* = 0.053), and elevated cardiac troponin I was observed in 3 and 2 patients, respectively. For nonhematological AEs, grade 1 to 2 fatigue occurred in 56.6% and 58.6% of patients, with grade 3 to 4 events in 3.8% and 1.7%, respectively. Grade 1 to 2 diarrhea was reported in 9.4% and 10.3%, with 1 grade 3 to 4 case in the SCRT-TNT-ICI group. Immune-related AEs mainly included hypothyroidism (grade 1 to 2, 11.3%), hyperthyroidism (grade 1 to 2, 9.4%), and dermatitis (grade 1 to 2, 5.7%; grade 3 to 4, 1.9%). One patient (1.9%) in the SCRT-TNT-ICI group developed immune-related enteritis, and another (1.9%) experienced elevated cardiac troponin I. No cases of immune-related pneumonia or hypophysitis were observed.

**Table 3.** Adverse events during neoadjuvant treatment in the mITT population

Adverse events	SCRT-TNT-ICI group (*n* = 53)		SCRT-TNT group (*n* = 58)		*P* value*
	Grade 1 to 2	Grade 3 to 4	Grade 1 to 2	Grade 3 to 4	
Any adverse events	40 (75.5%)	5 (9.4%)	44 (75.9%)	6 (10.3%)	0.999
Hematological					
Anemia	18 (34.0%)	3 (5.7%)	14 (24.1%)	4 (6.9%)	0.427
Leukopenia	34 (64.2%)	1 (1.9%)	42 (72.4%)	1 (1.7%)	0.408
Neutropenia	24 (45.3%)	0 (0.0%)	28 (48.3%)	1 (1.7%)	0.705
Febrile neutropenia	0 (0.0%)	0 (0.0%)	0 (0.0%)	1 (1.7%)	1.000
Thrombocytopenia	5 (9.4%)	0 (0.0%)	7 (12.1%)	0 (0.0%)	0.764
Alanine aminotransferase increased	16 (30.2%)	0 (0.0%)	11 (19.0%)	0 (0.0%)	0.190
Aspartate aminotransferase increased	24 (45.3%)	0 (0.0%)	19 (32.8%)	0 (0.0%)	0.242
Creatinine increased	0 (0.0%)	0 (0.0%)	1 (1.7%)	0 (0.0%)	1.000
Bilirubin increased	3 (5.7%)	0 (0.0%)	2 (3.4%)	0 (0.0%)	0.668
Hypothyroidism	6 (11.3%)	0 (0.0%)	1 (1.7%)	0 (0.0%)	0.053
Cardiac troponin I increased	3 (5.7%)	0 (0.0%)	2 (3.4%)	0 (0.0%)	0.668
Nonhematological					
Fatigue	30 (56.6%)	2 (3.8%)	34 (58.6%)	1 (1.7%)	1.000
Abdominal pain	11 (20.8%)	2 (3.8%)	16 (27.6%)	1 (1.7%)	0.670
Anal pain	12 (22.6%)	0 (0.0%)	18 (31.0%)	1 (1.7%)	0.291
Tenesmus	40 (75.5%)	2 (3.8%)	44 (75.9%)	3 (5.2%)	0.817
Diarrhea	5 (9.4%)	1 (1.9%)	6 (10.3%)	0 (0.0%)	1.000
Constipation	6 (11.3%)	0 (0.0%)	8 (13.8%)	1 (1.7%)	0.587
Nausea	35 (66.0%)	2 (3.8%)	36 (62.1%)	2 (3.4%)	0.688
Anorexia	30 (56.6%)	0 (0.0%)	36 (62.1%)	0 (0.0%)	0.569
Vomiting	11 (20.8%)	1 (1.9%)	10 (17.2%)	1 (1.7%)	0.648
Peripheral sensory neuropathy	30 (55.6%)	1 (1.9%)	33 (56.9%)	1 (1.7%)	1.000
Hand–foot syndrome	10 (18.9%)	0 (0.0%)	11 (19.0%)	0 (0.0%)	1.000
Dermatitis	8 (15.1%)	1 (1.9%)	3 (5.2%)	0 (0.0%)	0.066
Immune-related adverse events					
Any adverse events	9 (16.9%)	1 (1.9%)	0 (0.0%)	0 (0.0%)	0.000
Hypothyroidism	6 (11.3%)	0 (0.0%)	0 (0.0%)	0 (0.0%)	0.010
Hyperthyroidism	5 (9.4%)	0 (0.0%)	0 (0.0%)	0 (0.0%)	0.022
Cardiac troponin I increased	1 (1.9%)	0 (0.0%)	0 (0.0%)	0 (0.0%)	0.478
Dermatitis	3 (5.7%)	1 (1.9%)	0 (0.0%)	0 (0.0%)	0.049
Enteritis	1 (1.9%)	0 (0.0%)	0 (0.0%)	0 (0.0%)	0.478

mITT, modified intention-to-treat

* *P* values were calculated using Fisher’s exact test for the comparison of any-grade (Grade 1 to 4) adverse events between the 2 groups. For comparisons involving zero-event cells, Fisher’s exact test was still applied.

Postoperative complications (Clavien–Dindo graded) in the SCRT-TNT-ICI versus SCRT-TNT groups were as follows: pelvic sepsis, 1 case each (grade IIIa versus II); wound infection, 0 versus 2 cases (grades I and II); anastomotic leak, 1 (grade II) versus 2 (both grade II); anastomotic stenosis, 0 versus 2 (both grade I); intestinal obstruction, 8 cases (1 grade I and 7 grade II) versus 4 (3 grade I and 1 grade II). Thirty-day readmissions occurred in 2 SCRT-TNT-ICI patients (for obstruction and tenesmus) and 6 SCRT-TNT patients (all for adjuvant chemotherapy). No 60-d readmissions were due to surgical complications in either group.

## Discussion

The prospective phase II Neo-STAR trial evaluated adding ICIs to SCRT-TNT in LARC. Despite a lack of statistical significance (*P* = 0.052), the SCRT-TNT-ICI regimen showed a promising pCR rate of 45.3% versus 27.6% with SCRT-TNT, alongside a manageable safety profile. These findings support the potential of this approach and merit further investigation in larger trials.

In recent years, in the field of neoadjuvant therapy for LARC, several phase II to III studies have demonstrated that sequential administration of 2 to 6 cycles of chemotherapy combined with ICIs following SCRT can significantly increase the pCR rate. For instance, in studies such as UNION, TORCH, STELLAR II, and SPRING-01, the pCR rates in the combination ICIs groups ranged from 39.8% to 59.2%, significantly higher than the 15.3% to 32.7% observed in the control groups [12,15,18,19]. The latest consensus guidelines for ICIs in LARC strongly advocate for a preoperative regimen of SCRT combined with ICIs and 2 to 6 cycles of CAPOX, based on this cumulative evidence [25]. In the present study, although the intergroup difference did not reach statistical significance, potentially due to the limited sample size, the results are consistent with the trend reported in previous research. Notably, the MPR rate was significantly superior in the SCRT-TNT-ICI group compared to the SCRT-TNT group (50.9% versus 31.0%, *P* = 0.033), further indicating a clear advantage of the combination therapy in promoting tumor regression. Existing evidence suggests that patients who achieve either pCR or MPR have similar long-term survival outcomes, both of which are significantly better than those of patients who do not achieve such responses. For example, a study by Vecchio et al. [26] reported 5-year disease-free survival (DFS) rates of 96%, 90%, 74%, and 52% for patients with complete response, major response, minor response, and poor response, respectively. Similar to prior studies, our Neo-STAR study suggests that adding ICIs to the SCRT-TNT regimen may enhance tumor regression.

Compared to LCRT combined with ICIs, the SCRT combined with ICIs regimen shows a more favorable trend in short-term efficacy. A recently published meta-analysis indicated that, compared to ICI combined with LCRT the sequential approach of chemotherapy-ICIs followed by SCRT significantly improved the overall complete response rate (51% versus 36%) and the pCR rate (48% versus 30%), with both differences being statistically significant (*P* < 0.001) [27]. The underlying mechanism may be related to the ability of hypofractionated radiotherapy to more effectively enhance the expression of immunogenic signals (such as adhesion molecules, death receptors, stress-induced ligands, heat shock proteins, etc.), thereby eliciting a stronger antitumor immune response [28–30]. In contrast, conventional fractionated radiotherapy may not induce an immune activation effect of comparable intensity.

While research on neoadjuvant chemoradiotherapy combined with ICIs for LARC continues to grow, evidence regarding long-term survival remains limited. The NRG-GI002 trial is currently the only study to report such outcomes. It found that although short-term efficacy, measured by pCR rate (31.9% versus 29.4%), did not differ significantly, the pembrolizumab group achieved a significantly higher 3-year OS rate compared to the control group (95% versus 87%; *P* = 0.04) [31]. In the Neo-STAR study, although the combination ICIs group showed numerical advantages in 3-year OS (93.3% versus 87.9%) and PFS (78.7% versus 71.9%), neither reached statistical significance, suggesting that the improvement in pathological response did not directly translate into a survival benefit. Possible reasons for this are analyzed as follows: on the one hand, the median follow-up time in this study is still relatively short (less than 3 years), while LARC patients generally have a long OS period, meaning that survival differences may not yet be fully apparent; on the other hand, the survival benefit from tumor regression in individual patients may be diluted in inter-group comparisons, failing to manifest as a statistically significant difference. Similar phenomena have also been observed in previous LARC studies. For example, in the FORWARC study [32], although the FOLFOX-radiotherapy group had a significantly higher pCR rate than the chemotherapy-alone group (27.5% versus 6.6%), and patients who achieved pCR had a significantly better 10-year DFS (*P* < 0.0001), there was no statistical difference in 10-year OS or DFS between the 2 groups. In summary, whether the addition of ICIs to neoadjuvant chemoradiotherapy can bring long-term survival benefits to LARC patients remains to be established through more prospective studies and longer follow-up data.

In the Neo-STAR trial, subgroup analysis revealed that patients with the following characteristics were more likely to benefit from the SCRT-TNT-ICI regimen: age ≤60 years, a PD-L1 CPS ≥1, cT3 stage, and positive EMVI. Age demonstrated an inconsistent relationship with ICI efficacy across trials, largely attributable to heterogeneous cutoffs (45, 50, 60, and 65 years). In Neo-STAR, patients aged ≤60 years derived significant benefit (pCR: 50% versus 20%), as did younger patients in UNION and SPRING-01 (65-year threshold) and NECTAR (50-year threshold) [12,13,18]. In contrast, TORCH (45-year cutoff) showed the opposite trend, with patients ≥45 years achieving a higher complete response rate (60.8%) than those <45 years (26.3%) [15]. PD-L1 CPS status similarly showed variable predictive value—while CPS ≥1 was generally associated with improved responses in the previous studies [12,17], the CPS <1 subgroup in our trial failed to achieve statistical significance, likely due to limited statistical power, and whether CPS ≥1 or ≥2 represents the optimal threshold remains unresolved. Tumor stage exhibited a more consistent pattern, with combination therapy conferring pronounced pathological regression in earlier T-stage disease. The Neo-STAR trial demonstrated a significant pCR advantage in T3 patients (54.3% versus 25.0%; *P* = 0.012), a finding corroborated by UNION, TORCH, and SPRING-01, whereas the benefit in T4 patients was markedly more modest [12,15,18]. EMVI status, by contrast, yielded contradictory results: Neo-STAR and SPRING-01 trials suggested enhanced efficacy in EMVI-positive patients [18], whereas UNION and TORCH trials indicated greater benefit in the EMVI-negative subgroup [12,15]. Given these discrepancies, prospective trials with uniform stratification will help further characterize these observations.

The safety analysis revealed that grade 3 to 4 AEs occurred in 9.4% of patients in the SCRT-TNT-ICI group and 10.3% in the SCRT-TNT group, with no fatal events reported. Anemia was the most common severe event, accounting for 5.7% and 6.9% of cases in the respective groups, without a significant intergroup difference in hematological toxicity. Immune-related AEs were predominantly grade 1 to 2 hypothyroidism, which was markedly more prevalent in the SCRT-TNT-ICI group (11.3%) than in the SCRT-TNT group (1.7%). This toxicity profile led to postponed surgeries, a lengthened radiotherapy–surgery interval, and the withdrawal of a few patients in the SCRT-TNT-ICI group. Finally, the risk of postoperative complications was similar between the groups, suggesting no additional safety liability from the inclusion of ICIs.

This study had some limitations. Firstly, as a single-center, phase II RCT, the sample size was relatively small, which also accounts for the observation that the differences between the 2 groups in our study approached but did not reach statistical significance. Secondly, there was an imbalance in baseline age between the 2 groups in this study. However, through PSM analysis, we found that the MPR of the SCRT-TNT-ICI group was still significantly higher than that of the SCRT-TNT group, and the pCR rate was also higher than that of the control group, approaching a statistically significant difference. This is similar to the statistical results of the mITT population before matching. Thus, we thought that the imbalance in baseline age did not affect the results of the entire group of patients. Thirdly, there was a small sample size of patients with deficient mismatch repair/MSI-H. In the future, we can conduct relevant research on a broader population to explore the efficacy of the SCRT-TNT-ICI. Lastly, survival data (PFS/OS) are still immature, as the follow-up period has not yet reached 3 years; continued follow-up will clarify long-term outcomes.

## Conclusions

In conclusion, this phase II study indicates that combining SCRT-TNT with tislelizumab may provide potential benefits for tumor regression in patients with LARC. These preliminary findings require further validation in large-scale, multicenter, phase III RCTs. Future stratified research could help identify patients most likely to benefit, thereby supporting a more personalized treatment approach. Additionally, within the current radioimmunotherapy framework, optimizing the radiotherapy target volume, such as node-sparing radiotherapy, may further enhance outcomes while reducing treatment-related toxicity.

## Ethical Approval

This study involving human participants was approved by the Ethics Committee of the Fourth Hospital of Hebei Medical University (Approval number: 2021104). Written informed consent was obtained from all participants prior to enrollment. The study was conducted in compliance with the Declaration of Helsinki and Good Clinical Practice guidelines.

## Data Availability

The datasets generated and analyzed in this study are available from the corresponding author (48902034@hebmu.edu.cn) upon reasonable request.

## References

[1] Bahadoer RR, Dijkstra EA, Van Etten B, Marijnen CAM, Putter H, Kranenbarg EM-K, Roodvoets AGH, Nagtegaal ID, Beets-Tan RGH, Blomqvist LK, et al. Short-course radiotherapy followed by chemotherapy before total mesorectal excision (TME) versus preoperative chemoradiotherapy, TME, and optional adjuvant chemotherapy in locally advanced rectal cancer (RAPIDO): A randomised, open-label, phase 3 trial. Lancet Oncol. 2021;22(1):29–42. 33301740 10.1016/S1470-2045(20)30555-6

[2] Conroy T, Bosset J-F, Etienne P-L, Rio E, François É, Mesgouez-Nebout N, Vendrely V, Artignan X, Bouché O, Gargot D, et al. Neoadjuvant chemotherapy with FOLFIRINOX and preoperative chemoradiotherapy for patients with locally advanced rectal cancer (UNICANCER-PRODIGE 23): A multicentre, randomised, open-label, phase 3 trial. Lancet Oncol. 2021;22(5):702–715. 33862000 10.1016/S1470-2045(21)00079-6

[3] Jin J, Tang Y, Hu C, Jiang L-M, Jiang J, Li N, Liu W-Y, Chen S-L, Li S, Lu N-N, et al. Multicenter, randomized, phase III trial of short-term radiotherapy plus chemotherapy versus long-term chemoradiotherapy in locally advanced rectal cancer (STELLAR). J Clin Oncol. 2022;40(15):1681–1692. 35263150 10.1200/JCO.21.01667 PMC9113208

[4] Petrelli F, Trevisan F, Cabiddu M, Sgroi G, Bruschieri L, Rausa E, Ghidini M, Turati L. Total neoadjuvant therapy in rectal cancer: A systematic review and meta-analysis of treatment outcomes. Ann Surg. 2020;271(3):440–448. 31318794 10.1097/SLA.0000000000003471

[5] André T, Shiu K-K, Kim TW, Jensen BV, Jensen LH, Punt C, Smith D, Garcia-Carbonero R, Benavides M, Gibbs P, et al. Pembrolizumab in microsatellite-instability–high advanced colorectal cancer. N Engl J Med. 2020;383(23):2207–2218. 33264544 10.1056/NEJMoa2017699

[6] Cercek A, Lumish M, Sinopoli J, Weiss J, Shia J, Lamendola-Essel M, El Dika IH, Segal N, Shcherba M, Sugarman R, et al. PD-1 blockade in mismatch repair–deficient, locally advanced rectal cancer. N Engl J Med. 2022;386(25):2363–2376. 35660797 10.1056/NEJMoa2201445 PMC9492301

[7] Chalabi M, Fanchi LF, Dijkstra KK, Van Den Berg JG, Aalbers AG, Sikorska K, Lopez-Yurda M, Grootscholten C, Beets GL, Snaebjornsson P, et al. Neoadjuvant immunotherapy leads to pathological responses in MMR-proficient and MMR-deficient early-stage colon cancers. Nat Med. 2020;26(4):566–576. 32251400 10.1038/s41591-020-0805-8

[8] Chalabi M, Verschoor YL, Tan PB, Balduzzi S, Van Lent AU, Grootscholten C, Dokter S, Büller NV, Grotenhuis BA, Kuhlmann K, et al. Neoadjuvant immunotherapy in locally advanced mismatch repair–deficient colon cancer. N Engl J Med. 2024;390(21):1949–1958. 38838311 10.1056/NEJMoa2400634

[9] Corrò C, Dutoit V, Koessler T. Emerging trends for radio-immunotherapy in rectal cancer. Cancer. 2021;13(6):1374. 10.3390/cancers13061374 PMC8003099 33803620

[10] Stary V, Wolf B, Unterleuthner D, List J, Talic M, Laengle J, Beer A, Strobl J, Stary G, Dolznig H, et al. Short-course radiotherapy promotes pro-inflammatory macrophages via extracellular vesicles in human rectal cancer. J Immunother Cancer. 2020;8(2): Article e000667. 32817359 10.1136/jitc-2020-000667 PMC7437887

[11] Lin Z, Cai M, Zhang P, Li G, Liu T, Li X, Cai K, Nie X, Wang J, Liu J, et al. Phase II, single-arm trial of preoperative short-course radiotherapy followed by chemotherapy and camrelizumab in locally advanced rectal cancer. J Immunother Cancer. 2021;9(11): Article e003554. 34725214 10.1136/jitc-2021-003554 PMC8562535

[12] Lin ZY, Zhang P, Chi P, Xiao Y, Xu XM, Zhang AM, Qiu XF, Wu JX, Yuan Y, Wang ZN, et al. Neoadjuvant short-course radiotherapy followed by camrelizumab and chemotherapy in locally advanced rectal cancer (UNION): Early outcomes of a multicenter randomized phase III trial. Ann Oncol. 2024;35(10):882–891. 38964714 10.1016/j.annonc.2024.06.015

[13] Yang Z, Gao J, Zheng J, Han J, Li A, Liu G, Sun Y, Zhang J, Chen G, Xu R, et al. Efficacy and safety of PD-1 blockade plus long-course chemoradiotherapy in locally advanced rectal cancer (NECTAR): A multi-center phase 2 study. Signal Transduct Target Ther. 2024;9(1):56. 38462629 10.1038/s41392-024-01762-y PMC10925604

[14] Rahma OE, Yothers G, Hong TS, Russell MM, You YN, Parker W, Jacobs SA, Colangelo LH, Lucas PC, Gollub MJ, et al. Use of total neoadjuvant therapy for locally advanced rectal cancer: Initial results from the pembrolizumab arm of a phase 2 randomized clinical trial. JAMA Oncol. 2021;7(8):1225–1230. 34196693 10.1001/jamaoncol.2021.1683 PMC8251652

[15] Xia F, Wang Y, Wang H, Shen L, Xiang Z, Zhao Y, Zhang H, Wan J, Zhang H, Wang Y, et al. Randomized phase II trial of immunotherapy-based total neoadjuvant therapy for proficient mismatch repair or microsatellite stable locally advanced rectal cancer (TORCH). J Clin Oncol. 2024;42(28):3308–3318. 38950321 10.1200/JCO.23.02261

[16] Yang Y, Pang K, Lin G, Liu X, Gao J, Zhou J, Xu L, Gao Z, Wu Y, Li A, et al. Neoadjuvant chemoradiation with or without PD-1 blockade in locally advanced rectal cancer: A randomized phase 2 trial. Nat Med. 2025;31(2):449–456. 39762418 10.1038/s41591-024-03360-5

[17] Xiao W-W, Chen G, Gao Y-H, Lin J-Z, Wu X-J, Luo H-L, Lu Z-H, Wang Q-X, Sun R, Cai P-Q, et al. Effect of neoadjuvant chemoradiotherapy with or without PD-1 antibody sintilimab in pMMR locally advanced rectal cancer: A randomized clinical trial. Cancer Cell. 2024;42(9):1570–1581.e4. 39094560 10.1016/j.ccell.2024.07.004

[18] Tian F, Dai H, Sha D, Yu Y, Jing H, Sun C, Shang L, Liu Y, Feng R, Li J, et al. Total neoadjuvant treatment with short-course radiotherapy followed by sintilimab plus capecitabine–oxaliplatin versus short-course radiotherapy followed by capecitabine–oxaliplatin in patients with locally advanced rectal cancer (SPRING-01): A single-centre, open-label, phase 2, randomised controlled trial. Lancet Oncol. 2025;26(8):1043–1054. 40645192 10.1016/S1470-2045(25)00286-4

[19] Tang Y, Li H-Y, Wei L-C, Li N, Zhang W-J, Lu Y-F, Deng F-Y, Xu T-Z, Shuai J-C, Lei Z-F, et al. Short-course-based TNT with or without PD-1 inhibitor for pMMR locally advanced rectal cancer: Phase 2 results of a randomized trial (STELLAR II). Med. 2025;6(11): Article 100807. 40845854 10.1016/j.medj.2025.100807

[20] International Commission on Radiation Units and Measurements. *Prescribing, recording, and reporting photon-beam intensity-modulated radiation therapy* (IMRT). Oxford (UK): Oxford University Press; 2010. Report No.: ICRU Report 83.

[21] Lee NY, Lu JJ. *Target volume delineation and field setup: a practical guide for conformal and intensity-modulated radiation therapy*. Berlin, Heidelberg: Springer Berlin Heidelberg; 2013.

[22] US Department of Health and Human Services, Common Terminology Criteria for Adverse Events (CTCAE) Version 5.0. Bethesda (MD): National Institutes of Health, National Cancer Institute; 2017. NIH Publication No. 18-1559.

[23] Dindo D, Demartines N, Clavien P-A. Classification of surgical complications: A new proposal with evaluation in a cohort of 6336 patients and results of a survey. Ann Surg. 2004;240(2):205–213. 15273542 10.1097/01.sla.0000133083.54934.ae PMC1360123

[24] National Comprehensive Cancer Network. Management of Immunotherapy-Related Toxicities. Plymouth Meeting (PA) National Comprehensive Cancer Network; 2021.

[25] Yang Z, Xia F, Li J, Jin J, Wang G, Lin G, Li X, Wu A, Zhang T, Zhang W, et al. Consensus guidance of immune checkpoint inhibitors in locally advanced rectal cancer. Gut. 2026;gutjnl–2025–336979: 10.1136/gutjnl-2025-336979.41748160

[26] Vecchio FM, Valentini V, Minsky BD, Padula GDA, Venkatraman ES, Balducci M, Miccichè F, Ricci R, Morganti AG, Gambacorta MA, et al. The relationship of pathologic tumor regression grade (TRG) and outcomes after preoperative therapy in rectal cancer. Int J Radiat Oncol Biol Phys. 2005;62(3):752–760. 15936556 10.1016/j.ijrobp.2004.11.017

[27] Wang Y, Liu Y, Guan X, Liu X, Tang Y, Zhang W-W, Du C-X, Zou S-M, Zhou H-T, Liang J-W, et al. Neoadjuvant immunotherapy and chemoradiotherapy for mismatch repair proficient locally advanced rectal cancer: A systematic review and meta-analysis. Radiother Oncol. 2025;211: Article 111073. 40759282 10.1016/j.radonc.2025.111073

[28] Demaria S, Guha C, Schoenfeld J, Morris Z, Monjazeb A, Sikora A, Crittenden M, Shiao S, Khleif S, Gupta S, et al. Radiation dose and fraction in immunotherapy: One-size regimen does not fit all settings, so how does one choose? J Immunother Cancer. 2021;9(4): Article e002038. 33827904 10.1136/jitc-2020-002038 PMC8031689

[29] Yin L, Xue J, Li R, Zhou L, Deng L, Chen L, Zhang Y, Li Y, Zhang X, Xiu W, et al. Effect of low-dose radiation therapy on abscopal responses to hypofractionated radiation therapy and anti-PD1 in mice and patients with non-small cell lung cancer. Int J Radiat Oncol Biol Phys. 2020;108(1):212–224. 32417411 10.1016/j.ijrobp.2020.05.002

[30] Zhao X, Wang B, Zhuang Y, Du S, Zeng Z. Single high-dose irradiation-induced iRhom2 upregulation promotes macrophage antitumor activity through cGAS/STING signaling. Int J Radiat Oncol Biol Phys. 2023;116(5):1150–1162. 36792017 10.1016/j.ijrobp.2023.02.013

[31] George TJ, Yothers G, Rahma OE, Hong TS, Russell MM, You YN, Parker W, Jacobs SA, Lucas PC, Colangelo LH, et al. Long-term results from NRG-GI002: A phase II clinical trial platform using total neoadjuvant therapy (TNT) in locally advanced rectal cancer (LARC). J Clin Oncol. 2023;41(4):7.36343307

[32] Zhang J, Chi P, Shi L, Cui L, Gao J, Li W, Wei H, Cheng L, Huang Z, Cai G, et al. Neoadjuvant modified infusional fluorouracil, leucovorin, and oxaliplatin with or without radiation versus fluorouracil plus radiation for locally advanced rectal cancer: Updated results of the FOWARC study after a median follow-up of 10 years. J Clin Oncol. 2025;43(6):633–640. 39671537 10.1200/JCO-24-01676

