# Supplementary material for: Short-Course Radiotherapy-Based Total Neoadjuvant Therapy plus Tislelizumab for Locally Advanced Rectal Cancer (Neo-STAR): Early Outcomes of a Randomized Phase II Trial
Source: Cancer Commun (Lond). 2026 Jul 23;46:0041. doi: 10.34133/cancomm.0041 (PMC13392285; doi:10.34133/cancomm.0041)
Supplement: Supplementary 1 — Tables S1 and S2 [file cancomm.0041.f1.pdf]

Supplementary materials for

Short-course Radiotherapy-based Total Neoadjuvant Therapy plus Tislelizumab for Locally Advanced Rectal Cancer (Neo-STAR): Early Outcomes of a Randomized Phase II Trial

Fengpeng Wu[1, †], Xuhua Hu[2, †], Baokun Li[2, †], Jianfeng Zhang[2, †], Guanglin Wang[2, †], Guangquan An[2], Bin Yu[2], Haiyan Fan[3], Hongqing Ma[2], Botian Zhao[1], Zhihan Li[2], Bo Gao[2], Ming Liu[1], Xuan Wang[1], Dan Liu[1], Jitao Hu[2], Hui Liu[2], Youqiang Liu[2], Feifei Wang[2], Juan Zhang[2], Jun Feng[2], Xiaoran Wang[2], Zesong Meng[2], Zhenya Zhang[2], Zheng Li[2], Jingyi Sun[1], Shihao Liu[4], Na Wang[5], Jing Han[6], Wenbo Niu[2, *], Chaoxi Zhou[2, *], Linlin Xiao[1, *], Guiying Wang[2, 4, *]

[1]Department of Radiation Oncology, the Fourth Hospital of Hebei Medical University, Shijiazhuang, Hebei 050011, P. R. China.

[2]The Second Department of Surgery, the Fourth Hospital of Hebei Medical University, Shijiazhuang, Hebei 050011, P. R. China.

[3]Department of Gastroenterology, the Fourth Hospital of Hebei Medical University, Shijiazhuang, Hebei 050011, P. R. China.

[4]Department of General Surgery, the Second Hospital of Hebei Medical University, Shijiazhuang, Hebei 050004, P. R. China.

[5]Cancer Institute, the Fourth Hospital of Hebei Medical University, Shijiazhuang, Hebei 050011, P. R. China.

[6]Department of Medical Oncology, the Fourth Hospital of Hebei Medical University, Shijiazhuang, Hebei 050011, P. R. China.

[*]Address correspondence to:

Guiying Wang, the Second Department of Surgery, the Fourth Hospital of Hebei Medical University, Shijiazhuang, 050011, Hebei, P. R. China, e-mail: wangguiying@hebmu.edu.cn

Linlin Xiao, Department of Radiation Oncology, the Fourth Hospital of Hebei Medical University, Shijiazhuang, Hebei 050011, P. R. China, e-mail: 48902034@hebmu.edu.cn

Chaoxi Zhou, the Second Department of Surgery, the Fourth Hospital of Hebei Medical University, Shijiazhuang, 050011, Hebei, P. R. China, e-mail: 48101085@hebmu.edu.cn

Wenbo Niu, the Second Department of Surgery, the Fourth Hospital of Hebei Medical University, Shijiazhuang, 050011, Hebei, P. R. China, e-mail: 47900878@hebmu.edu.cn

[†]These authors have contributed equally as first authors.

Study Protocol Summary

Brief Summary
This study was a single-center, prospective, open-label, randomized controlled clinical study, and the purpose of this study was to compare the pathological complete response rate (pCR) of patients with locally advanced rectal cancer treated with short-course radiotherapy sequential Tislelizumab combined with CAPOX (capecitabine plus oxaliplatin; group A) versus short-course radiotherapy sequential CAPOX (group B). A minimum of 100 patients with locally advanced rectal cancer was required for this study based on sample size calculations. Patients were randomly assigned to the experimental group (group A) and the control group (group B) in a 1:1 ratio.

Schema and treatment plan

Subjects in group A were treated according to the following treatment plan:
Standard short-course radiotherapy (SCRT): A total radiation dose of 25 Gy was delivered in 5 fractions (from day 1 to 5)
Sequential treatment period: After resting for 3-7 days following completion of SCRT, patients were treated with 4 cycles of CAPOX (oxaliplatin 130 mg/m^2 intravenously, day 1; capecitabine 1000 mg/m^2, twice daily, day 1-14) and an additional intravenous infusion of 200 mg Tislelizumab on the first day of each cycle of CAPOX.
Surgery: After 3 weeks of the completion of neoadjuvant therapy, total mesorectal excision (TME) surgery was performed.
Postoperative adjuvant chemotherapy: whether postoperative chemotherapy was implemented mainly depended on the patient's wishes, with 2 cycles of CAPOX with or without Tislelizumab given to willing cases.

Subjects in group B were treated according to the following treatment plan:
Standard SCRT: A total radiation dose of 25 Gy was delivered in 5 fractions (from day 1 to 5)
Sequential treatment period: After resting for 3-7 days following completion of SCRT, patients were treated with 4 cycles of CAPOX (Oxaliplatin 130 mg/m^2 intravenously, day 1; Capecitabine 1000 mg/m^2, twice daily, day 1-14).
Surgery: After 3 weeks of the completion of neoadjuvant therapy, TME surgery was performed.
Postoperative adjuvant chemotherapy: whether postoperative chemotherapy was implemented mainly depended on the patient's wishes, with 2 cycles of CAPOX given to willing cases.

Rules for dose modification
When any of the following situations occur in the subjects, the dosage of administration can be adjusted:
1) Absolute Neutrophil Count (ANC) $< 0.5 \times 10^9/L$;
2) Febrile neutropenia (FN);
3) ANC count is between $0.5 \times 10^9/L$ and $1.0 \times 10^9/L$, and PLT count is between $25 \times 10^9/L$ and $50 \times 10^9/L$;
4) Platelet (PLT) count is less than $25 \times 10^9/L$, or bleeding, or requiring blood transfusion;
5) Grade ≥3 anemia;
6) Grade ≥3 diarrhea;
7) Grade ≥3 nausea/vomiting;
8) Grade ≥3 hand-foot syndrome;
9) Grade ≥3 neurotoxicity;
10) Grade ≥3 immune-related toxic side effects;
11) Other toxicities that are judged by the investigators to require dosage adjustment.

Up to 2 dose reductions were allowed. If more reduction is needed, the subject was withdrawn from the study. If the subject withdraws from the treatment due to intolerable toxicity, follow-up should be conducted until the toxicity is relieved.
Once the dose is reduced, all subsequent administrations should maintain the reduced dose or further reduce the dose if necessary.
If multiple toxic reactions occur during a treatment cycle, the highest level of toxicity reaction should be used as the parameter for dose adjustment. If toxicity can be clearly attributed to one drug, the researcher can choose to reduce only the dose of one cytotoxic drug.
If it is considered in the best interest of the subject, the researcher can decide to use supportive measures for treatment/prevention instead of dose reduction in the next treatment course.
For those toxic reactions that the researcher considers unlikely to cause serious or life-threatening events and will not delay or interrupt the treatment (such as hair loss, altered taste, etc.), the study drug should not be reduced or suspended.

The dose-adjustment schema for systemic therapeutic agents was predefined as follows: The starting doses were 130 mg/m² for oxaliplatin, 1000 mg/m² for capecitabine, and 200 mg for tislelizumab. For oxaliplatin, two dose-reduction levels were specified: dose level 1 at 98 mg/m² and dose level 2 at 65 mg/m². Similarly, capecitabine was reduced in two steps, from 1000 mg/m² to 750 mg/m² (dose level 1) and then to 500 mg/m² (dose level 2). No predefined dose-reduction levels were applied for tislelizumab. During the treatment period, if any adverse reactions caused by Tislelizumab occur, depending on the severity of the adverse reactions, the treatment can be either suspended or permanently discontinued without dose adjustment.

Measurement of the treatment effect

The pCR is defined as ypT0N0M0. The MPR is defined as TRG of 0 or 1.

The histopathological assessment of tumor regression followed the standards of the 8th Edition of the American Joint Committee on Cancer (AJCC). TRGs were assessed as follows: 0, no remaining viable cancer cells (complete response); 1, a single cell or small groups of cancer cells (moderate response); 2, residual cancer outgrown by fibrosis (minimal response); 3, minimal or no tumor cell death, with extensive residual cancer (poor response).

The evaluation of clinical complete response (cCR) must meet all the following criteria: 1) enhanced pelvic Magnetic Resonance Imaging + Diffusion-Weighted Imaging (MRI+DWI) showing that the primary tumor disappeared or shrank significantly; 2) no suspicious lymph nodes on MRI; 3) no residual tumor at endoscopy or only a small residual erythematous ulcer or scar; 4) negative biopsies from the scar, ulcer, or former tumor location; 5) no palpable tumor detected by digital anal examination.

Adverse events (AEs) were closely monitored and actively managed during the SCRT process and the resting period thereafter, the entire preoperative systemic treatment, the resting period after the TME surgery, and the postoperative chemotherapy (the willing cases). AEs were assessed by National Cancer Institute Common Terminology Criteria for Adverse Events (NCI-CTCAE) Version 5.0.

PFS was defined as the time from randomization to disease progression, recurrence or death for any cause. OS was defined as the time from randomization to death for any cause.

Follow-up records during treatment

For the duration of operation, the time required to complete TME surgery and the amount of blood loss were recorded, and the impact of neoadjuvant therapy on the operation was observed. During the SCRT process, and the period of resting after SCRT, of the entire preoperative systemic treatment, of the resting after TME surgery, and of the postoperative chemotherapy (for willing cases), the occurrence of AEs in participants was closely monitored and actively managed.

Eligibility Criteria

- Patients or their family members agreed to participate in the study and signed the informed consent form;
- Patients ≥18 and ≤75 years old, male or female;
- Eastern Cooperative Oncology Group (ECOG) performance status of 0 or 1;
- Patients with histologically confirmed rectal adenocarcinoma;
- The clinical diagnosis of chest computed tomography (CT), abdominal and pelvic enhanced MRI was $T_{1-2}N_{+}M_{0}$ and $cT_{3-4}N_{any}M_{0}$ (the T and N stage was based on pelvic enhanced MRI+DWI, M stage was determined by liver enhanced MRI+DWI and chest CT, and if necessary, positron emission tomography-computed tomography was used);
- The distance between the lower edge of the tumor and the anal edge is less than or equal to 10 cm;
- No history of immune system diseases;
- No history of immunodeficiency, including HIV positive;
- No history of other malignancies;
- No history of myocarditis;
- No history of severe cardiovascular and cerebrovascular diseases;
- No history of thyroid dysfunction;
- No history of liver and kidney diseases;
- No history of mental illness, no history of Infectious diseases;
- No history of organ transplantation or allogeneic bone marrow transplantation;
- No history of other systemic diseases other than the above diseases;
- Voluntarily accept the neoadjuvant treatment scheme of radiotherapy, sequential chemotherapy/chemotherapy combined with immunotherapy;
- Can swallow pills normally;
- Patients must not have undergone any prior anti-cancer treatment, including radiotherapy, chemotherapy, surgical resection, or traditional Chinese medicine therapy, etc., for the tumor.
- Patients must be candidates for and have a plan for definitive surgical resection following neoadjuvant

therapy.

Ineligibility Criteria

- Patients who do not meet the above inclusion criteria;
- Documented history of allergy to study drugs, including any component of tislelizumab, capecitabine, oxaliplatin and other platinum drugs;
- Patients who need to be treated with corticosteroid (dose equivalent to prednisone of >10 mg/day) or other immunosuppressive agents within 2 weeks prior to study drug administration; Major surgery or severe trauma within 4 weeks before the first use of the study drug;
- Severe infection (CTCAE >2) occurred within 4 weeks before the first use of the study drug; Baseline chest imaging revealed active pulmonary inflammation, signs and symptoms of infection within 14 days prior to the first use of the study drug, or oral or intravenous antibiotic therapy, except for prophylactic use of antibiotics;
- Female patients who are pregnant or breastfeeding;
- Patients who refuse to sign informed consent by themselves or their authorized persons;
- Patients with poor cognitive ability, unable to answer questions, unable to fill in questionnaires or with mental disorders;
- Patients were considered unsuitable for the study by the investigator.

Supplementary Table S1. Baseline characteristics of eligible patients after propensity score matching

Characteristics	SCRT-TNT-ICI group (*n* = 27)	SCRT-TNT group (*n* = 27)
**Sex**		
Male	19 (70.4%)	17 (63.0%)
Female	8 (29.6%)	10 (37.0%)
**Age, year**		
Median (range)	58 (36-74)	60 (36-75)
**Age category**		
≤60 years	15 (55.6%)	13 (50.0%)
>60 years	12 (44.4%)	13 (50.0%)
**ECOG PS**		
0	16 (59.3%)	16 (59.3%)
1	11 (40.7%)	11 (40.7%)
**PD-L1 CPS**		
<1	7 (25.9%)	9 (33.3%)
≥1	14 (51.9%)	13 (48.1%)
Unknown	6 (22.2%)	5 (18.5%)
**MMR status**		
pMMR or MSI-L or MSS	27 (100.0%)	27 (100.0%)
**Clinical T stage**		
cT2	0 (0.0%)	2 (7.4%)
cT3	21 (77.8%)	16 (59.3%)
cT4	6 (22.2%)	9 (33.3%)
**Clinical N stage**		
N0	0 (0.0%)	2 (7.4%)
N1	8 (29.6%)	5 (18.5%)
N2	19 (70.4%)	20 (74.1%)
**Clinical stage**		
II	2 (7.4%)	0 (0.0%)
III	25 (92.6%)	27 (100.0%)
**Distance from primary tumor to anal verge**		
≤5 cm	16 (59.3%)	17 (63.0%)
5-10 cm	11 (40.7%)	10 (37.0%)
**EMVI**		
Positive	13 (48.1%)	12 (44.4%)
Negative	14 (51.9%)	15 (55.6%)
**MRF**		
Positive	15 (55.6%)	11 (40.7%)
Negative	12 (44.4%)	16 (59.3%)

Abbreviations: ECOG PS, Eastern Cooperative Oncology Group Performance Status; PD-L1 CPS, programmed death-ligand 1 combined positive score; MMR, mismatch repair; dMMR, deficient mismatch repair; pMMR, proficient mismatch repair; EMVI, extramural vascular invasion; MRF, mesorectal fascia.

Supplementary Table S2. The postoperative pathological outcomes in the PSM population

**PSM population**	**SCRT-TNT-ICIs group (*n* = 27)**	**SCRT-TNT group (*n* = 27)**
pCR, *n* (%; 95% CI)	15 (55.6; 35.4%-74.5%)	8 (29.6; 13.8%-49.4%)
MPR, *n* (%; 95% CI)	17 (63.0; 42.5%-80.6%)	9 (33.3; 16.5%-54.0%)

PSM, propensity score-matching; pCR, pathological complete response; MPR, major pathological response; CI, confidence interval.
